# Experimental solubility of aripiprazole in supercritical carbon dioxide and modeling

**DOI:** 10.1038/s41598-023-40537-3

**Published:** 2023-08-17

**Authors:** Eslam Ansari, Bizhan Honarvar, Seyed Ali Sajadian, Zahra Arab Aboosadi, Mehdi Azizi

**Affiliations:** 1 Department of Chemical Engineering, Marvdasht Branch, Islamic Azad University, Marvdasht, Iran; 2 Department of Chemical Engineering, Faculty of Engineering, University of Kashan, Kashan, 87317-53153 Iran; 3 South Zagros Oil and Gas Production, National Iranian Oil Company, Shiraz, 7135717991 Iran

**Keywords:** Medical research, Drug development

## Abstract

The solubility of compounds in supercritical carbon dioxide (SC-${\mathrm{CO}}_{2}$) has found crucial significance in the fabrication of micro/nano-scaled drugs. In this research, the solubility of Aripiprazole was measured in SC-${\mathrm{CO}}_{2}$ at various temperatures (308–338 K) and pressures (12–30 MPa). Moreover, the experimental solubility results were correlated with several semi-empirical models (Chrastil, Bartle et al., Kumar & Johnston, Menden-Santiago & Teja, Sodeifian et al., and Jouyban et al.) as well as the modified Wilson model. The molar fraction of the drug in SC-${\mathrm{CO}}_{2}$ varied in the range of $1.830\times {10}^{-6}$ to $1.036\times {10}^{-5}$. The solubility highly depended on the operating pressure and temperature. The Chrastil (0.994), Jouyban et al. (0.993) and Sodeifian et al. (0.992) models showed the highest consistency with the obtained values. Furthermore, self-consistency tests were performed on the solubility of Aripiprazole in SC-${\mathrm{CO}}_{2}$. The approximate total enthalpy (${\mathrm{\Delta H}}_{\mathrm{total}}$), vaporization enthalpy (${\mathrm{\Delta H}}_{\mathrm{vap}}$), and solubility enthalpy (${\mathrm{\Delta H}}_{\mathrm{sol}}$) were also calculated.

Aripiprazole (APZ) is a second-generation antipsychotic, known as a typical antipsychotic. This drug is effective in a wide range of psychotic disorders such as schizophrenia^1^. It can also serve as a mood stabilizer in the treatment of bipolar disorder^2–4^. APZ has been approved by the Food and Drug Administration (FDA) for the treatment of mixed episodes associated with bipolar disorder and acute manic. It appears that functional selectivity at D_2_ receptors may contribute to the antipsychotic effects of APZ^5–7^. As a typical antipsychotic compound, APZ selectively binds targeting serotonin and central dopamine D_2_ receptors which can be effective in the treatment of cognitive and negative symptoms of schizophrenia^1,7^. Based on previous studies, APZ can prevent the activation of microglia by reducing the inflammatory cytokines^8–10^. APZ can be used in the treatment of depression due to its effect on microglia activities and anti-inflammatory behavior. However, low bioavailability of Aripiprazole due to its poor aqueous solubility has significantly limited the development of APZ-based drugs and their therapeutic effects on depression^8,11^.

Drugs with poor water solubility often show poor oral bioavailability and limited absorption rate. The improvement of the absorption, solubility, and permeability of poorly water-soluble drugs is one of the major research topics^12^. The dissolution rate of the pharmaceutical compounds increases by decline of their particle size. Several conventional methods such as grinding, sieving, spray drying, and re-crystallization can be used to reduce the particle size. Each of these methods has their own drawbacks. In the last decade, supercritical fluids (SCF) technology has been employed as a micronization process to serve as an alternative to traditional methods. Researchers have used supercritical fluids as solvent or anti-solvent in the extraction processes, solution-enhanced dispersion, and solutions/suspensions rapid expansion methods^13–22^. In addition to its moderate critical point (304.1 K as temperature and 7.38 MPa as pressure), SC-CO_2_ enjoys non-polluting nature, non-flammability, non-explosiveness, and accessibility in high purity^15,23–26^. In the process of nanoparticle production, the drug solubility in supercritical fluid is the main parameter as it determines the feasibility of supercritical methods. This parameter also specifies the role of SCF as solvent, anti-solvent, or reaction medium^27,28^. The RESS-based processes are generally employed to prepare nanoparticle drugs with high SC-CO_2_ solubility, in contrast, anti-solvent procedures are suitable in the preparation of the drug with low solubility^29–32^. A wide range of drugs with different SC -${\mathrm{CO}}_{2}$ solubility levels have been recently examined^25,33–35^. Moreover, various approaches have been developed to measure the drug solubility in SC–${\mathrm{CO}}_{2}$, among which, gravimetric^36–39^, spectrometric^40–42^, chromatographic^43,44^, and miscellaneous^46^ methods can be mentioned. Modeling methods can also help to investigate the solubility of pharmaceuticals in SC–${\mathrm{CO}}_{2}$ with far lower time and costs and no need for complex equipment^13^.

Various mathematical models have been developed to assess the solubility of different compounds in SC–${\mathrm{CO}}_{2}$. These methods can be categorized into several groups including equation of state (EoS), empirical and semi-empirical models, expanded liquid models, square support vector machine (LS-SVM), and artificial neural network (ANN) techniques^45,47^. Empirical and semi-empirical models are capable of correlating the experimental solubility data to operating conditions such as temperature, pressure, and the ratio of the cosolvents to the supercritical solvent density. No need for pure solid properties is the greatest advantage of these models^23,46,47^. The EoS are classified into two categories: cubic EoS, such as the Peng-Robinson (PR)^50^ and the Soave- Redlich- Kowang (SRK)^51^, and non-cubic EoS. Activity coefficient-based models like modified Wilson models and universal quasi-chemical models (UNIQUAC) can be used to correlate the solubility data. The physicochemical properties of solid solutes are required in both EoS-based and activity coefficient models whose experimental measurement is a challenging and complex task. Therefore, some methods have been developed to determine the properties of solute molecules^48^. Empirical and semi-empirical models (Chrastil^49^, Bartle et al.^46^, Kumar and Johnston (K-J)^53^, Garlapati et al*.*^54^, Menden -Santiago & Teja (MST)^55^, Sodeifian et al.^33^, Jouyban et al.^56^) and expanded liquid models (universal quasi chemical, modified Wilson's model)^50,51^ have been utilized to determine the solubility of various drugs in SC–${\mathrm{CO}}_{2}$.

In this study, the APZ solubility in SC-${\mathrm{CO}}_{2}$ was experimentally assessed at various pressures (12–30 MPa) and temperatures (308–338 K). The results were correlated with several models including Chrasti, Bartle et al. (K-J), MST, Sodeifian et al. Jouyban et al. and modified Wilson's model. The accuracy of these models in the correlation of APZ solubility was explored by calculating and comparing average absolute relative deviation (AARD %) and adjusted correlation coefficient (${R}_{adj}$).

## Materials and methods

### Materials

A sample of APZ (form II) with CAS number of 9-12-129722 and purity of 99% was purchased from Tofigh Daru Pharmaceutical Company (Tehran, Iran). Carbon dioxide (CAS number 124-38-9) with a purity of 99.98% was also supplied from Oxygen Novin Company (Shiraz, Iran). Methanol (CAS number 67-56-1) with minimum purity of 99.9% was also provided from Merck (Germany).

### Physical and critical characteristics

The solubility of APZ in SC-${\mathrm{CO}}_{2}$ was quantified by thermodynamic investigations using appropriate group participation methods. The melting point (${\mathrm{T}}_{\mathrm{m}}$) was determined by DSC analysis while the boiling point (${\mathrm{T}}_{\mathrm{b}}$), critical pressure (${\mathrm{P}}_{\mathrm{c}}$), and critical temperature (${\mathrm{T}}_{\mathrm{c}}$) were evaluated by the Marrero and Gani contribution method^59^. To calculate these features, the molecular structure of APZ was broke down to 10CH_2_, 6CH (cyclic), 2C (cyclic), 2C-CL (cyclic), 2N (cyclic), 1C-O (cyclic), 1C-NH (cyclic), 1C, and 1O (cyclic). The molar volume (${V}_{S}$) and Grain Watson^52^, sublimation pressure (${\mathrm{P}}_{\mathrm{S}}$), and the corresponding modes of Ambrose-Walton^53^ factor (ω) were determined according to the Immirzi-Perini method^54^, as listed in Table 1.

**Table 1:** The solute Aripiprazole structure and the respective physic-chemical features.

Component	Formula	Mw (g.mol^−1^)	CAS Number	Structure						
APZ	C_23_H_27_CL_2_N_3_O_2_	448.39	129722-12-9							
Carbon dioxide	CO_2_	44.01	124-38-9							

Component	T_m_ (K)	T_b_ (K)	T_c_ (K)	P_c_ (bar)	ω	V_s_ (cm^3^.mol^−1^)	P_sub_ (bar)^e^			
							308.15 K	318.15 K	328.15 K	338.15 K
APZ	415.11^a^	744.93^b^	981.18^b^	6.07^b^	0.3189^c^	552.8^d^	2.28 × 10^−6^	6.90 × 10^−6^	1.97 × 10^−5^	5.33 × 10^−5^
CO_2_	–	–	304.18	73.8	0.225					

*T*_*b*_ boiling point, T_c_ critical point, *P*_*c*_ critical pressure, *ω* acentric factor, *V*_*s*_ solid molar volume, *T* temperature, *P*_*sub*_ sublimation pressure.

^a^Calculated with DSC analysis.

^b^Calculated with Group contribution^59^.

^c^Calculated with the Ambrose–Walton corresponding states method^53^.

^d^Calculated with Immirzi–Perini method^54^.

^e^Calculated with Grain-Watson method^52^.

### Experimental setup and solubility assessment

**Figure 1:**
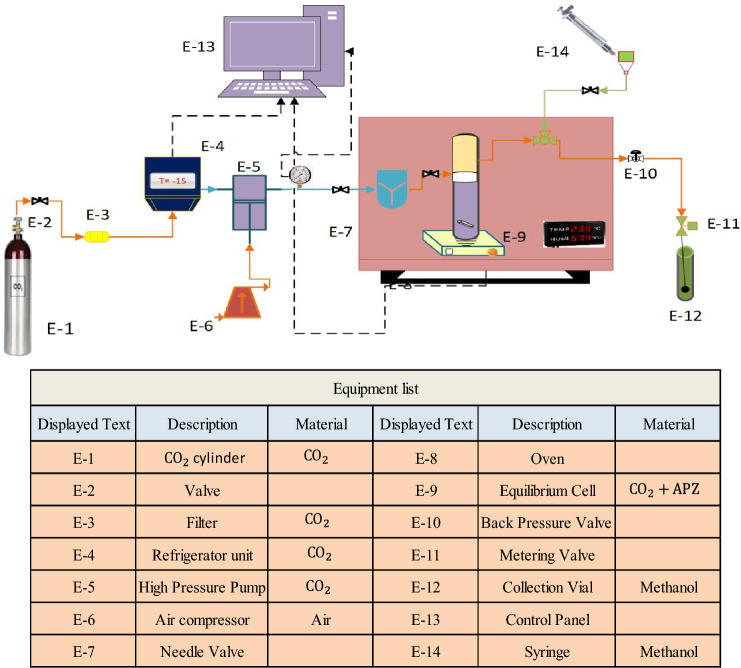
Schematic diagram of the SC-CO_2_ solubility measurement used in this research.

The experimental setup of this device includes a ${\mathrm{CO}}_{2}$ cylinder (E-1), valve (E-2), filter (E-3), refrigeration unit (E-4), high-pressure pump (E-5), air compressor (E-6), Needle valve (E-7), oven (Memmert) (E-8), equilibrium cell (E-9), back pressure valve (E-10), metering valve (E-11), collection vial (E-12), control panel (E-13), syringe (E-14), digital pressure transmitter (WIKA, Germany, code IS-0-3-2111), pressure gauge (WIKA, Germany, code EN 837-1), a digital thermometer, and 1.8-inch pipe and fittings (Fig. 1).

The high-pressure system was made of stainless steel 316. In a typical process, ${\mathrm{CO}}_{2}$ first passed through a 1 µm filter to be purified on its way to the refrigerator, at which, its temperature was reduced to – 15 °C for liquefaction. The pressure of liquid ${\mathrm{CO}}_{2}$ was then increased up to 12–30 MPa. Such a high pressure can be controlled through a reciprocating pump. APZ (1 g) and liquid ${\mathrm{CO}}_{2}$ were then mixed and homogenized by a magnetic stirrer (100 rpm) in a cell placed in an oven for 120 min. The static time, drug content, and purity were checked by some preliminary tests. At the end of the static time, 600 μL of saturated SC-${\mathrm{CO}}_{2}$ was loaded into the injection loop through a three-valve two-position valve. By opening the injection valve, the sample collected inside the vial was released with 5 ml of methanol which had been already loaded. Subsequently, the vial was washed by the syringe pump which injected 1 ml of methanol. The drug content of the obtained sample was evaluated by a spectrophotometer at a wavelength of 254 nm. A calibration curve was also used to estimate the concentration of the solutes. A set of standard solutions were obtained through diluting the stock solutions. Drug solubility in SC-${\mathrm{CO}}_{2}$ can be calculated at various pressures and temperatures using the following equations:

1 $${y}_{2}=\frac{{n}_{solute}}{{n}_{solute}+{n}_{{CO}_{2}}}$$

2 $${\mathrm{n}}_{\mathrm{solute}}=\frac{{\mathrm{C}}_{\mathrm{s}}\times {\mathrm{V}}_{\mathrm{s}}}{{\mathrm{M}}_{\mathrm{S}}},\mathrm{and}$$

3 $${\mathrm{n}}_{{\mathrm{CO}}_{2}}=\frac{{\mathrm{V}}_{\mathrm{l}}\times\uprho }{{\mathrm{M}}_{{\mathrm{CO}}_{2}}} ,$$

where ${\mathrm{n}}_{\mathrm{solute}}$ and ${\mathrm{n}}_{{\mathrm{CO}}_{2}}$ denote the number of moles of the solute and ${\mathrm{CO}}_{2}$, respectively, ${\mathrm{C}}_{\mathrm{S}}$ shows the solute concentration ($\mathrm{g}.{L}^{-1}$) based on the calibration curve. $\mathrm{Vs}(\mathrm{L})$ and $\mathrm{Vl}(\mathrm{L})$ represent the volumes of the sampling vial and collection ring, respectively. ${\mathrm{M}}_{\mathrm{S}}$ and ${\mathrm{M}}_{\mathrm{CO}2}$ stand for the molecular mass of the solute and ${\mathrm{CO}}_{2}$, respectively. Equation (4) expresses the equilibrium solubility of the solute (S) in SC-${\mathrm{CO}}_{2}$:

4 $$\mathrm{S}=\frac{\uprho \times {\mathrm{M}}_{\mathrm{solute}}\times {\mathrm{y}}_{2}}{{\mathrm{M}}_{{\mathrm{CO}}_{2}}\times \left(1-{\mathrm{y}}_{2}\right)} .$$

## Results and discussion

### Experimental data

**Figure 2:**
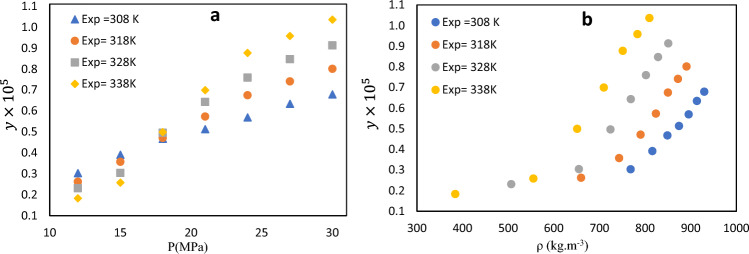
Experimental solubility of APZ in SC-CO_2_ at various pressures and temperatures. (**a**) Solubility according to pressure and (**b**) solubility according to density.

The solubility of APZ in SC-${\mathrm{CO}}_{2}$ was examined at different temperatures (308–338 K) and pressures (12–30 MPa). The measurements were carried out in triplicates to reduce the error. Data of APZ solubility in SC-CO_2_ including its mole fraction (y), density (ρ), solubility (S), and expanded uncertainty are also presented in Table 2. Accordingly, the highest APZ mole fraction $(1.036\times {10}^{-5}$) was detected at 338 K and 30 MPa whereas the lowest value ($1.830\times {10}^{-6}$) was recorded at 338 K and 12 MPa. The solubility showed an ascending trend with increasing the pressure at high temperatures. As the pressure rises, the density of SC-${\mathrm{CO}}_{2}$ increases which enhances the strength of the solvent. The solvent density and the vapor pressure of the solution are the main factors in the solubility enhancement. Based on Fig. 2, the solubility curve showed a crossover region. Temperature generally exhibited a dual effect on drug solubility in SC-CO_2_ under controlled SC-CO_2_ density and drug vapor pressure. The solubility of APZ in SC-CO_2_ decremented in the pressure range of 12–18 MPa by enhancing the temperature. At pressures above 18 MPa, the solubility rose with temperature elevation. The crossover region for APZ ranged from 12 to 18 MPa. At pressures lower than 18 MPa, the effect of density was predominant as the solubility increased by temperature reduction. However, at pressures above 18 MPa, solubility rose with temperature increment due to the predominance of the influence of the vapor pressure of the drug. The impact of temperature on carbon dioxide density and vapor pressure of solute was reported by several articles with similar values of the SC − CO_2_ pressure crossover region for Nystatin^55^, Clonazepam^56^ and famotidine^57^. These transitions can be attributed to temperature-induced density changes in carbon dioxide and vapor pressure changes in solutes. The crossover pressure was investigated in several articles, which proposed some methods to predict the crossover pressure region^58–60^. The crossover region varies depending on the critical properties of the solute, such as its sublimation pressure, sublimation enthalpy, partial molar enthalpy, and molar volume. Thus, the pressure range of 12–18 MPa was introduced as the crossover region for APZ drug (Fig. 2).

**Table 2:** The APZ solubility at distinctive operational conditions (12–30 MPa) and (308–338 K).

Temperature (K)^a^	Pressure (MPa)^a^	Density (kg.m^−3^)^b^	Y_2_ × 10^5^ (Mole Fraction)	Standard deviation of the mean, SD(ȳ) × (10^5^)	Expanded uncertainty × 10^6^	S × 10 (Solubility (g.l^−1^))
308	12	768.42	0.303	0.015	0.306	0.237
	15	816.06	0.391	0.026	0.526	0.325
	18	848.87	0.467	0.022	0.450	0.404
	21	874.4	0.513	0.034	0.688	0.457
	24	895.54	0.569	0.050	1.007	0.520
	27	913.69	0.634	0.064	1.287	0.591
	30	929.68	0.679	0.079	1.586	0.644
318	12	659.73	0.262	0.012	0.246	0.176
	15	743.17	0.357	0.023	0.466	0.270
	18	790.18	0.471	0.031	0.627	0.379
	21	823.7	0.573	0.050	1.007	0.481
	24	850.1	0.675	0.079	1.586	0.585
	27	872.04	0.741	0.072	1.448	0.659
	30	890.92	0.801	0.090	1.807	0.727
328	12	506.85	0.231	0.007	0.148	0.119
	15	654.94	0.304	0.021	0.425	0.203
	18	724.13	0.496	0.025	0.510	0.366
	21	768.74	0.643	0.048	0.969	0.504
	24	801.92	0.759	0.073	1.468	0.620
	27	828.51	0.847	0.086	1.729	0.716
	30	850.83	0.913	0.077	1.551	0.792
338	12	384.17	0.183	0.006	0.126	0.072
	15	555.23	0.258	0.017	0.344	0.146
	18	651.18	0.499	0.043	0.866	0.331
	21	709.69	0.699	0.056	1.129	0.506
	24	751.17	0.877	0.078	1.570	0.671
	27	783.29	0.958	0.095	1.910	0.764
	30	809.58	1.036	0.120	2.409	0.855

The experimental standard deviation of the mean (SD) were obtained by $SD\left(\overline{y }\right)=\frac{s\left({y}_{k}\right)}{\sqrt{n}}$ . n is the number of times each experimental data was measured (n = 3, in this work). Expanded uncertainty is U = k*u_*combined*_ and the relative combined standard uncertainty is defined as *u*_*combined*_*/y *=$\sqrt{\sum_{i=1}^{N}\,{(\mathrm{Pi\,u}(\mathrm{xi})/\mathrm{xi})}^{2}}$ in which u(xi)/xi is the relative standard uncertainty of each input estimate (*xi*) and *P*_*i*_ is known positive or negative number having negligible uncertainties.

^a^Standard uncertainty u are u(T) = 0.1 K; u(p) = 1 bar. Also, the relative standard uncertainties are obtained below 0.05 for mole fractions and solubility’s. The value of the coverage factor k = 2 was chosen on the basis of the level of confidence of approximately 95 percent. Data from the Span–Wagner equation of state.

### Semi-empirical models

Semi-empirical models such as Chrastil^49^, Bartle et al.^61^, K-J^62^, MST^63^, Sodeifian et al.^33^, and Jouyban et al.^64^ were used for the correlation of the solubility of APZ. Table 3 lists the equations of the semi-empirical models. Chrastil^49^ proposed an equation for the solid solutes based on the SCF density and absolute temperature (${\mathrm{a}}_{2}=\frac{{\Delta \mathrm{H}}_{\mathrm{t}}}{\mathrm{R}} )$, in which, the adjustable parameter of ${a}_{2}$ is a function of the total heat. R shows the global gas constant and ${\Delta \mathrm{H}}_{\mathrm{t}}$ represents the total heat of mixing. The vaporization enthalpy (${\Delta \mathrm{H}}_{\mathrm{vap}}$) can be determined by the model proposed by Bartle et al*.*^61^. According to the Hess' law, the solvation enthalpy $\left({\Delta \mathrm{H}}_{\mathrm{sol}}\right)$ can be defined as the difference between ${\Delta \mathrm{H}}_{\mathrm{t}}$ and ${\Delta \mathrm{H}}_{\mathrm{vap}}$. Sodeifian et al*.* proposed a semi-empirical model ${a}_{0}-{a}_{5}$ and introduced six adjustable parameters. In 1998, K-J^62^ presented a density-based semi-empirical model for the correlation of the solid solubility in SCF. They expressed the relationship of ${a}_{2}$ with ${\Delta \mathrm{H}}_{\mathrm{t}}$ through ${\Delta \mathrm{H}}_{\mathrm{t}}=\frac{{\mathrm{a}}_{2}}{\mathrm{R}}$. A simple linear equation is shown by MST model for consistency of solid solubility in SCF.

**Table 3:** The semi-empirical models exploited in the present study.

Model	Formula
Chrastil^49^	$\mathrm{ln}\,s={a}_{0}\mathrm{ln}\rho +{a}_{1}+\frac{{a}_{2}}{T}$
Bartle et al.^61^	$\mathrm{ln}\,\frac{{y}_{p}}{{\rho }_{ref}}={a}_{0}+ {a}_{1}\left(\rho -{\rho }_{ref}\right)+\frac{{a}_{2}}{T}$
K-J^62^	$\mathrm{ln}\,s={a}_{0}+{a}_{1}\rho +\frac{{a}_{2}}{T}$
MST^63^	$\mathrm{ln}\,\left({Y}_{2}P\right)={a}_{0}+{a}_{1}\rho +{a}_{2}T$
Sodeifian et al.^33^	$\mathrm{ln}\,{y}_{2}={a}_{0}+\left({a}_{1}+{a}_{2}\rho \right)\mathrm{ln}\rho +\frac{{a}_{3}}{T}+{a}_{4}\mathrm{ln}\left(\rho T\right)$
Jouyban et al.^64^	$\mathrm{ln}\,{y}_{2}={a}_{0}+{a}_{1}\rho +{a}_{2}{P}^{2}+{a}_{3}PT+\frac{{a}_{4}T}{P}+{a}_{5}\mathrm{ln}\left(\rho \right)$

Semi-empirical models of Chrastil^49^, Sodeifian et al.^33^, K-J^62^, MST^63^, Bartle et al.^33^, and Jouyban et al.^64^ have three, six, three, three, three, and six parameters, respectively. The mentioned models were used from the Simulated Annealing algorithm for optimization. The adjustable parameters of the relevant statistical measures were obtained in terms of AARD% and ${R}_{adj}$ for the ${\mathrm{CO}}_{2}$-APZ binary system using the density-based models as listed in Table 4.

**Table 4:** Diverse parameters of APZ—CO_2_ binary system, as obtained using models proposed by Chrastil, Bartle et al. Kumar and Johnston, MST, Sodeifian et al. and Jouyban et al.

Model	${a}_{0}$	${a}_{1}$	${a}_{2}$	${a}_{3}$	${a}_{4}$	${a}_{5}$	AARD%	${R}_{adj}$
Chrastil^49^	4.8	− 3608.9	− 24	–	–	–	7.90	0.994
Bartle et al*.*^61^	10.7	0.008	5861.49	–	–	–	10.73	0.971
KJ^62^	− 4.5	0.2	3697.7	–	–	–	5.90	0.991
MST^63^	− 8265.9	2.8	11.9	–	–	–	9.30	0.981
Sodeifian et al*.*^33^	179.73	− 1.3	− 17.225	0.017	− 0.015	− 8309.61	5.89	0.992
Jouyban et al*.*^64^	− 20,722.88	2.093	− 4.570	0.088	72.423	291.170	4.39	0.993

The average absolute relative deviation (AARD %) was used to assess the precision of the models:

5 $$AARD\%=\frac{100}{{N}_{t}-Z}\sum_{i=1}^{{N}_{t}}\frac{\left|{y}_{2}^{calc}-{y}_{2}^{exp}\right|}{{y}_{2}^{exp}}$$

In the above equation, Z represents the number of adjustable parameters of each model, ${N}_{t}$ shows the number of data points in each set, and ${y}_{2}$ denotes the mole fraction solubility. The correlation coefficient adjusted by ${\mathrm{R}}_{\mathrm{adj}}$ is defined as follows:

6 $${R}_{adj}=\sqrt{\left|{R}^{2}-\left(Q\left(1-{R}^{2}\right)/\left(N-Q-1\right)\right)\right|}$$

While the correlation coefficient is represented by ${R}^{2}$, the number of data points in each set is shown by N. Q also denotes the number of independent variables in each equation.

**Figure 3:**
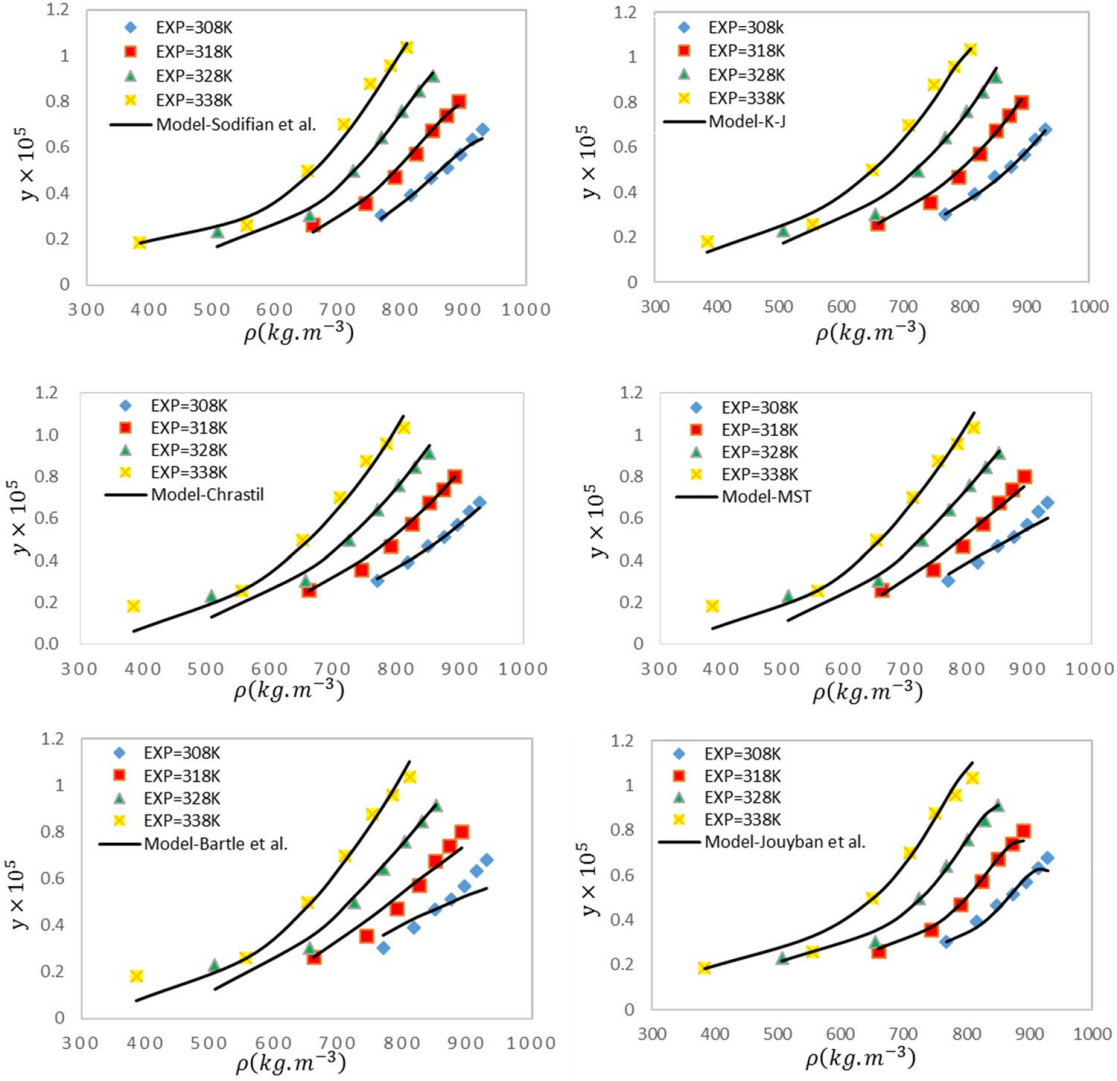
A comparison of experimental (points) and modeled (lines) values of APZ solubility based on semi-empirical models at different temperatures.

The AARD% values were 7.90, 10.73, 5.90, 9.30, 5.89, and 4.39 for Chrastil, Bartle et al., K-J, MST, Sodeifian et al., and Jouyban et al., respectively. The models proposed by Jouyban et al. and Sodeifian et al. showed the best performance in predicting the solubility of APZ with respective AARD% values of 4.39 and 5.89%. Jouyban et al. model exhibited the best correlation compared to others. The linear equation of Jouyban et al. is generally more suitable for predicting the solubility of this type of drug compared to the model proposed by Bartle et al. Other semi-empirical models offered acceptable predictive accuracies. The results also revealed the higher precision of the Chrastil model in predicting the solubility data with ${\mathrm{R}}_{\mathrm{adj}}$=0.994. Figure 3 compares the experimental solubility with those calculated by the density-based models.

**Figure 4:**
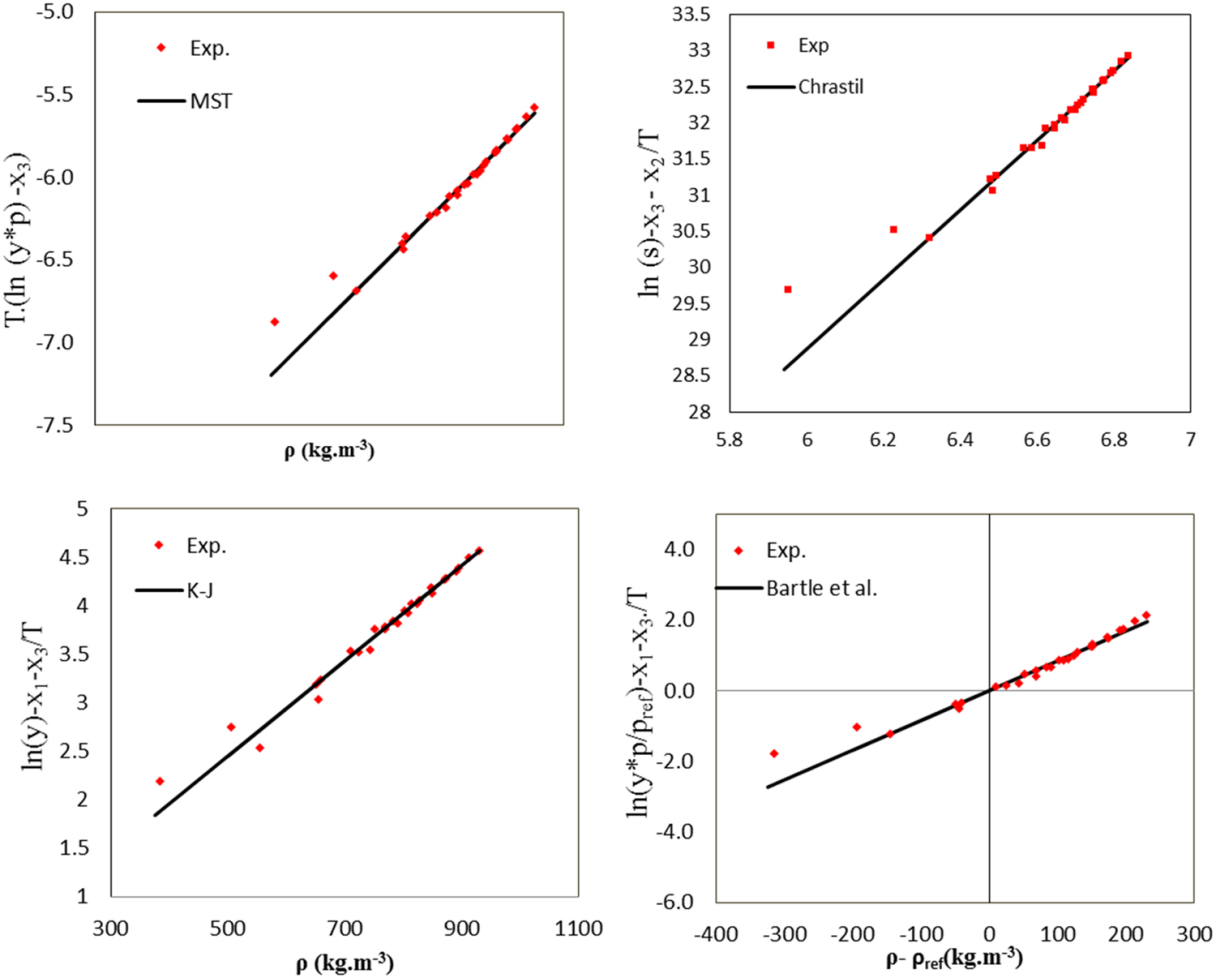
The self-consistency results obtained for four semi-empirical models. The lines suggest the linearity of the models.

Figure 4 demonstrates the self-consistency of experimental data of APZ solubility with Chrastil, Bartle et al*.*, MST, and K-J models. The model is acceptable in self-consistency tests if all the solubility data obtained at different temperatures are located on the $45-\mathrm{degree}$ line. The test results of the mentioned semi-empirical models suggest the consistency of the measured solubility values.

Table 5 lists the calculated enthalpy for APZ in SC-${\mathrm{CO}}_{2}$. The Chrastil model shows the approximate total heat of $30\mathrm{ KJ}.{\mathrm{mol}}^{-1}$. Based on Bartle’s model, the enthalpy of vaporization was $(48.73\mathrm{ KJ}.{\mathrm{mol}}^{-1})$. Solvation heat (${\Delta \mathrm{H}}_{\mathrm{sol}}$) was equal to $18.73\mathrm{ KJ}.{\mathrm{mol}}^{-1}$ based on the difference between ${\Delta \mathrm{H}}_{\mathrm{vap}}$ and ${\Delta \mathrm{H}}_{\mathrm{t}}$.

**Table 5:** The vaporization (ΔH_vap_), approximated total (T_total_), and solvation (ΔH_sol_) enthalpy for APZ.

Compound	ΔH_total_ $(\mathrm{KJ}.{\mathrm{mol}}^{-1}$)^a^	ΔH_vap._ ($\mathrm{KJ}.{\mathrm{mol}}^{-1}$)^b^	ΔH_sol._ ($\mathrm{KJ}.{\mathrm{mol}}^{-1}$)^c^
APZ	30.00	48.73	− 18.73

^a^Calculated by the Chrastil's model^49^.

^b^Calculated by the Bartle et al. model^33^.

^c^Calculated by the difference between the ΔH_vap_ and ΔH_total_.

### Modified Wilson model

Since the solid solubility in the supercritical phase is very small, we can assume to be at infinite dilution condition. Consequently, the activity coefficient of the solid solute is the one at infinite dilution (${\gamma }_{2}^{\infty }$) and the density of the solution is that of the pure solvent. Therefore, the solubility equation is obtained:

7 $${\mathrm{y}}_{2}=\frac{1}{{\upgamma }_{2}^{\infty }}\mathrm{exp}\left[\frac{{-\Delta \mathrm{H}}_{2}^{\mathrm{f}}}{\mathrm{R}}\left(\frac{1}{\mathrm{T}}-\frac{1}{{\mathrm{T}}_{\mathrm{m}}}\right)\right]$$

${-\Delta \mathrm{H}}_{2}^{\mathrm{f}}$ is the enthalpy of fusion and T_m_ is the melting point temperature of the solid solute.

Gibbs excess energy is defined according to the following formula for the binary system. Wilson’s model has two variable parameters $({\lambda {\prime}}_{12}and{\lambda {\prime}}_{21}$) which are the difference of intermolecular interaction energies of the molar volume of supercritical carbon dioxide. Moreover, ${\vartheta }_{1 }and {\vartheta }_{2}$ are dependent values due to the low solubility of the solute in the SC-CO_2_ where ${\vartheta }_{1 }and {\vartheta }_{2}$ are the molar volumes of the SCF (expanded liquid) and the solid solute respectively. The following equation can be used to determine the activity coefficient:

8 $$\mathrm{ln}\,{\gamma }_{2}^{\infty }=1-{\Lambda }_{12}-\mathrm{ln}{\Lambda }_{21}$$

${\vartheta }_{1 }\mathrm{and }{\vartheta }_{2}$ can be defined under infinite dilution conditions:

9 $${\Lambda }_{12}={\vartheta }_{2}{\rho }_{cl}{\rho }_{r}exp\left(-\frac{{\lambda {^\prime}}_{12}}{{T}_{r}}\right)$$

10 $${\Lambda }_{21}=\frac{1}{{\vartheta }_{2}{\rho }_{cl}{\rho }_{r}}exp\left(-\frac{{\lambda {^\prime}}_{21}}{{T}_{r}}\right)$$

${\rho }_{r}$ is the reduced density of the solvent (SCF) equal to $\rho$ /${\rho }_{cl}$, where ${\rho }_{cl}$ is its critical density and the dimensionless energies of interaction are as follow:

11 $${\lambda {\prime}}_{12}=\frac{{\lambda }_{12}}{R{T}_{C1}}$$

12 $${\lambda {\prime}}_{21}=\frac{{\lambda }_{21}}{R{T}_{C1}}$$

A linear equation can be defined between molar volume and reduced density to capture the effect of high pressure on the model:

13 $${\vartheta }_{2}=\alpha {\rho }_{r}+\beta$$

${{\lambda {\prime}}_{12},\lambda {\prime}}_{21}, \alpha ,\mathrm{ and }\beta$ were obtained by the model.

**Figure 5:**
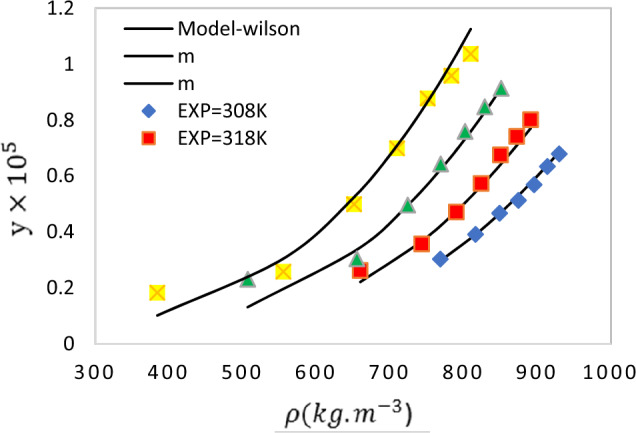
Experimental data (point) and calculated (line) solubility of APZ in SC-CO_2_ based on the modified Wilson model.

Using the extended liquid theory, the modified Wilson model was utilized for optimization of the parameters of the model of APZ solubility in SC-CO_2_. Table 6 summarizes the parameters of the modified Wilson model ($\alpha ,\beta ,{\lambda {\prime}}_{12},{\lambda {\prime}}_{21}$). A comparison of experimental and modeled data (Fig. 5) confirmed the accuracy of the modified Wilson model. Based on Table 6, ${\lambda {\prime}}_{21}$ is smaller than ${\lambda {\prime}}_{12}$ as also reported in previous studies^27,34,50,51,65^.

**Table 6:** Modified Wilson model parameters for solubility of APZ in SC-CO_2_.

$\mathrm{\rm A}$	$\mathrm{\rm B}$	${\mathrm{\lambda {\prime}}}_{12}$	${\mathrm{\lambda {\prime}}}_{21}$	AARD%	R_adj_
− 0.00000787	0.0000452	− 2.78033	16.66676	6.82	0.985

## Conclusion

APZ solubility was evaluated at different pressures (12, 15, 18, 21, 24, 27, and 30 MPa) and temperatures (308, 318, 328, and 338 K). The molar fraction of APZ in SC-CO_2_ varied from $1.83\times {10}^{-6}$ to $1.036\times {10}^{-5}.$ The lowest and highest molar fractions of APZ were detected at a constant temperature of 338 K and pressures of 12 and 30 MPa, respectively. Six semi-empirical models (Sodeifian et al., Jouyban et al., Chrastil, Bartle et al., MST, K-J), and an extended liquid theory (modified Wilson model) were employed for the correlation of the experimental solubility data. The precision of the models was explored in terms of AARD% and ${\mathrm{R}}_{\mathrm{adj}}$. Accordingly, the modified Wilson model (AARD% = 6.82) and the semi-empirical models of Chrastil (AARD% = 7.90), Bartle et al. (AARD% = 10.73), Jouyban (AARD% = 4.39), MST (AARD% = 9.30), Kumar Johnston (AARD% = 5.90), Sodeifian et al*.* (AARD% = 5.89), Jouyban et al*.,* and Sodeifian et al*.* with six adjustable parameters showed the best correlation among density-based models, reflecting the ability of this model to correlate solubility data. Such satisfactory correlation results of the semi-empirical models also show the self-consistency of the experimental findings. The models of Chrastil and Bartle et al. were also applied to determine the enthalpy of vaporization and solvation.

## Data Availability

The datasets used and/or analyzed during the current study are available upon reasonable request from the corresponding author.

## List of symbols

*AAR*D%: Percent AARD, Eq. (5), (Table 4)
${M}_{CO2}$: CO2 molecular weight, Eq. (3)
${M}_{S}$: Solute molecular weight, Eq. (2)
MST: Mendez-Santiago–Teja
${n}_{CO2}$: Mole of CO2, Eqs. (1), and (3)
${n}_{Solute}$: Mole of solute, Eqs. (1), and (2)
*P*_*c*_: Critical pressure, (Table 1)
${P}_{sub}$: Sublimation pressure (bar), (Table 1)
R: Gas constant universal (J.(mol K)^-1^)
${R}_{adj}$: Adjusted correlation coefficient, Eq. (6)
S: Solubility in equilibrium state (g.L^-1^), Eqs. (4)–(5)
T: Temperature (K)
${T}_{m}$: Melting point (K), (Table 1)
${T}_{b}$: Boiling point (K), (Table 1)
${T}_{C}$: Critical temperature (K), (Table 1)
${V}_{S}$: Solid molar volume (m^3^.mol^-1^), (Table 1)
VdW2: Two-parameter Van der Waals mixing rule
${V}_{I}$ (*L*): Sampling vial (L), Eq. (3)
${V}_{S}$ (*L*): Represent the volume (L), Eq. (2)
S: Solubility in equilibrium state, Eq. (4)
$${a}_{2}{a}_{0}$$: Parameter, (Tables 3, 4)
$${a}_{0}-{a}_{5}$$: Parameter, (Tables 3, 4)
${\Delta H}_{2}^{f}$: Enthalpy of fusion
${\Delta H}_{SOL}$: Solubility enthalpy, (Table 5)
${\Delta H}_{vap}$: Vaporization enthalpy, (Table 5)
${\Delta H}_{t}$: Total heat, (Table 5)
${\gamma }_{2}^{\infty }$: Infinite dilution, Eq. (7)
$\alpha ,\beta ,{\lambda }_{12},{\lambda }_{21}$: Parameters of the modified Wilson model, Eqs. (9, 10, 13), (Table 6)
${y}_{2}$: Mole fraction, Eq. (1)

## Greek symbols

y: Mole fraction
ρ: Density (kg.m^−3^)
ω: Acentric factor

## Subscripts

*i*, *j*: Component
2: Solute

## Superscripts

cal: Calculated
exp: Experimental
s: Solute
